# Impacts of decline harvest of country food on nutrient intake among Inuit in Arctic Canada: impact of climate change and possible adaptation plan

**DOI:** 10.3402/ijch.v75.31127

**Published:** 2016-07-05

**Authors:** Renata Rosol, Stephanie Powell-Hellyer, Hing Man Chan

**Affiliations:** 1 Department of Biology, University of Ottawa, Ottawa, ON, Canada; 2 School of Health Sciences, University of Northern British Columbia, Prince George, BC, Canada

**Keywords:** Inuit Health Survey, climate change, country food, food security, diet composition, nutrients

## Abstract

**Background:**

The pervasive food insecurity and the diet transition away from local, nutrient-rich country foods present a public health challenge among Inuit living in the Canadian Arctic. While environmental factors such as climate change decreased the accessibility and availability of many country food species, new species were introduced into regions where they were previously unavailable. An adaptation such as turning to alternate country food species can be a viable solution to substitute for the nutrients provided by the declined food species. The objective of this study was to estimate the impact on nutrient intake using hypothetical scenarios that current commonly harvested country foods were reduced by 50%, and were replaced with alternate or new species.

**Methods:**

Data collected during the 2007–2008 Inuit Health Survey from 36 Canadian Arctic communities spanning Nunavut, the Inuvialuit Settlement Region and Nunatsiavut were used.

**Results:**

A 50% decline in consumption of fish, whale, ringed seals and birds (the food that was reported to be in decline) resulted in a significant decrease in essential nutrient intake. Possible substitute foods were identified but some nutrients such as zinc and especially vitamin D were most often found lacking in the alternative diet.

**Conclusions:**

If the alternative species are not available or feasible, more expensive and less nutritionally dense store-bought foods may be sought. Given the superior quality of country foods and their association with food security, and Inuit cultural health and personal identity, developing skills and awareness for adaptation, promoting regional sharing networks, forming a co-management agency and continuing nutritional monitoring may potentially preserve the nutritional integrity of Inuit diet, and in turn their health and cultural survival.

The importance of country foods to the overall physical and mental health, and cultural well-being of Inuit is widely recognized and acknowledged (1–6). Hunting, harvesting and sharing of country food plays an integral part in social cohesion and cultural continuity for Inuit communities, and country food continues to be at the centre of Inuit identity and well-being (6). Country foods are nutrient-dense, contribute to higher dietary quality (4,7,8) and are associated with greater food security (9). Studies have shown that on days when country food was consumed, there were higher intakes of protein and vitamins A and C, and a lower intake of refined carbohydrates, saturated fat and sodium (2,5,10). A study of an indigenous population of Western Alaska found that this relatively high latitude population maintained their mean serum vitamin D concentration throughout the year through the use of locally harvested foods, specifically fish (11). They found that those individuals (mostly youths) who consumed less vitamin D–rich local fish were more likely to have low serum vitamin D. Wild-harvested country foods which include land animals, birds, fish, sea mammals and berries are rich in antioxidants, omega-3 fatty acids, monounsaturated fatty acids and protein, and are high in micronutrients such as iron, riboflavin, zinc, copper, magnesium, potassium, selenium, thiamine, niacin and vitamins A, D, E, B6 and B12 (2,10,12). Similar to other inhabitants of Arctic regions, such as the Alaska Native populations (13,14), Inuit are undergoing nutrition transition where they continue to consume country foods while increasingly rely on highly processed and expensive market foods shipped from the south (1,2,7,8). Although it is widely accepted that the benefit of country foods far outweigh the benefits of available market foods, there are significant challenges that Inuit must overcome in order to purchase healthier market foods and access traditional species.

It has been shown that Inuit suffer disproportionately from food insecurity compared to the rest of Canada (15–17). The results from the Inuit Health Survey showed that adults living in Nunavut, ISR and Nunatsiavut experienced high prevalence of food insecurity, at 68.8, 43.3 and 45.7%, respectively (16,17) compared to just 9.2% for the Canadian national average (18). Food insecure adults had a reduced consumption of country foods that led to a significantly lower intake of energy, vitamin C, iron, zinc and vitamin D (5). Among the Alaska Natives, 12.1% of households were food insecure as of 2012 (19), and similar to Canada, Indigenous people represented a larger proportion of the food insecurity burden. Currently, many Inuit are unable to access safe, sufficient, nutritionally adequate and socially acceptable food (6). Low income, high cost of hunting equipment and gas, insufficient quantity and compromised quality of market foods along with climate-related changes to the surrounding environment depict a multitude of barriers that may impede a person's ability to access healthy food (6,15,20–22). With limited access to sufficient and affordable nutritious food, healthy eating is difficult to achieve and increases the risk of diet-sensitive chronic diseases (2,5,23,24).

Local hunters and elders residing in the Canadian Arctic have long observed what scientific evidence has shown to date, that parts of Canada's north has experienced a general warming in temperature over the past 50 years, with some parts of the Arctic experiencing a temperature increase in excess of 2°C (25–27). Effects of climate-related changes are affecting the Inuit traditional way of life across the Canadian north. For example, with unpredictable weather patterns and stronger winds it has become more dangerous for Inuit to travel along the ice which they use to access their hunting grounds. The warmer temperatures cause sea ice to take longer to form in the fall and cause it to break up earlier in the spring decreasing the length of the hunting season. Other effects of climate change include coastal erosion, continued permafrost thaw, reduction in sea ice thickness and snow cover and changes in the availability, distribution and health of some Arctic wildlife and plant species (20,25,28–31). Inuit relying on traditional, locally harvested foods are especially vulnerable to the effects of climate change with far-reaching consequences to the nutritional composition of their diet and overall well-being. Adaptation will be necessary to cope with the impacts of climate change on the health of Inuit. An adaptation strategy of shifting to alternate or new traditional food species has historically been an important approach to ensuring food security, and will continue to be a viable solution in addressing the potential loss of key nutrients in Inuit diet (26). However, the impacts of such changes in diet composition of nutrient intake and diet quality are not known.

The objective of this study is to model the impacts of nutrient intake based on a hypothetical scenario of having commonly harvested country foods reduced by 50%, and replaced with alternate or new species. The hypothesis is that it is feasible to use alternative wildlife species to substitute for declining food species in the current Inuit diet without affecting the diet quality. The goal of the study is to provide evidence for policy makers in wildlife management and nutrition to increase the regional preparedness for climate change to promote healthy diet.

## Material and methods

### Study area and survey sample

**Fig. 1:**
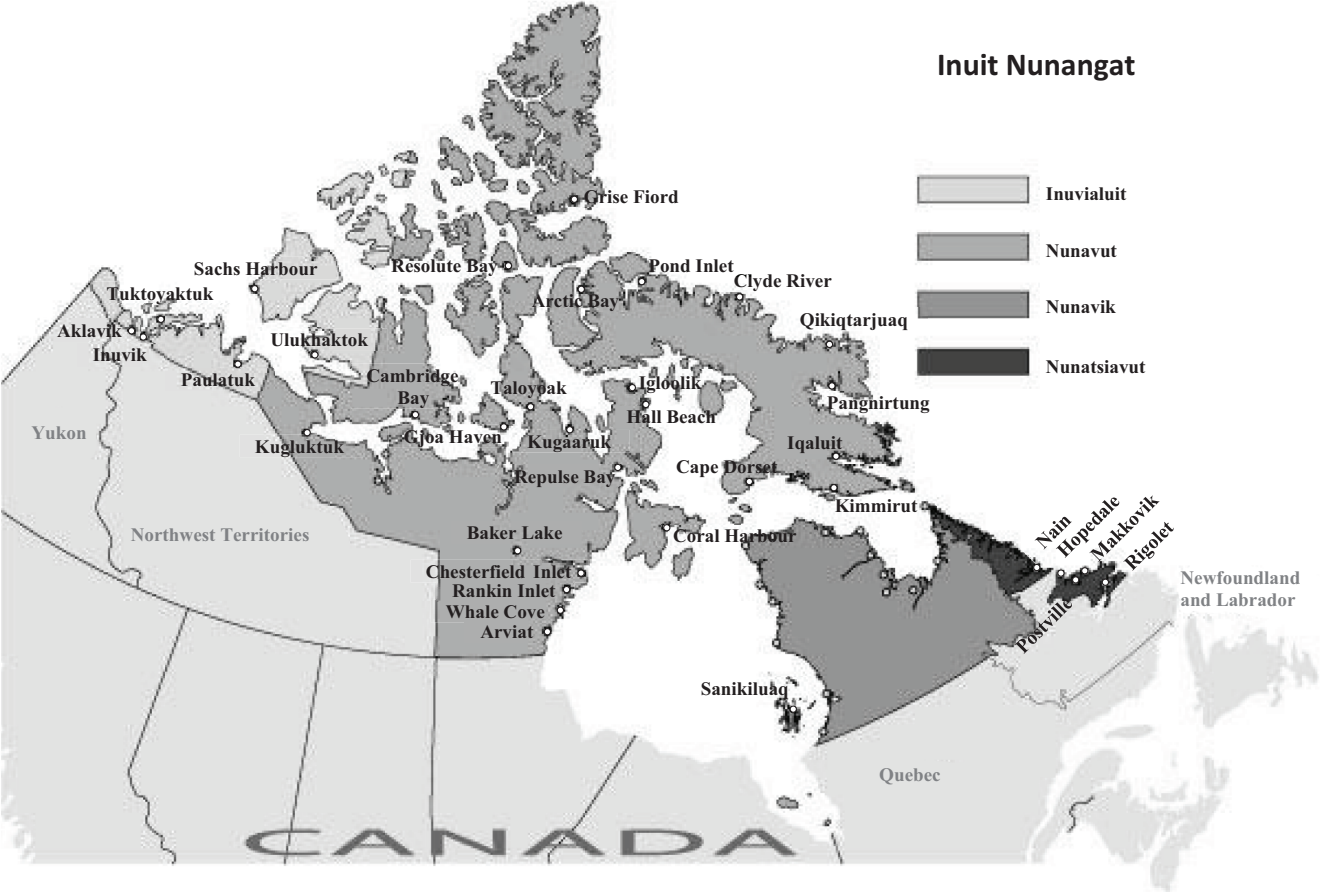
Map of the Inuvialuit Settlement Region, Nunavut and Nunatsiavut showing 36 communities that participated in the Adult Inuit Health Survey in 2007 and 2008. (Adapted and used with permission from Inuit Tapiriit Katanami (2009/07/28), Inuit regions of Canada: www.itk.ca/sites/default/files/InuitNunaat_Basic.pdf)

As part of the International Polar Year (IPY) activities, a cross-sectional health survey of the Inuit population was conducted across the Canadian Arctic in the summer and fall of 2007 and 2008. Thirty-three coastal communities and 3 non-coastal communities of the 3 jurisdictions which included the Inuvialuit Settlement Region in Northwest Territories, Nunavut (comprised of 3 regions: Kitikmeot, Kivalliq and Baffin) and Nunatsiavut in Northern Labrador, took part in the survey (Fig. 1). The communities were located between the latitude of 54°10′N and 76°25′N.

Stratified random sampling was carried out by trained research staff where communities were strata and homes were randomized using either a computerized random generation of numbers or a random digit table. All non-pregnant, self-identified Inuit adults 18 years of age and older were eligible to participate. Adults were recruited using community housing lists.

### Participatory research and ethics

The survey was developed with the participation of members of the steering committee of each jurisdiction. The steering committees included members from the government health agencies responsible for public health in each jurisdiction, community representatives, land-claim organizations and the principal investigators. Scientific research licenses were obtained from the Nunavummi Qaujisaqtulirijikkut (Nunavut Research Institute) and the Aurora Research Institute-Aurora College (Inuvik, Northwest Territories). The Nunatsiavut review board waived the requirement for a license given the extensive consultations that took place. A certificate of ethical acceptability was obtained from McGill University, and the University of Northern British Columbia. Consent forms, questionnaires and the DVD (consent form content presented orally and visually) were translated into different Inuit dialects appropriate for the regions surveyed, and all participants signed a consent form.

### Dietary assessments

Trained bilingual (English and Inuit dialects) interviewers administered 24 hour-record recalls in face-to-face interviews. The 24 hour-record recall completed by each participant was designed to capture the relative contribution of country food to the nutrient requirement of the participants in the regions of ISR, Nunavut and Nunatsiavut. For all food items, the participant was asked to quantify usual serving size using the food models and pictures provided. Food models (32) included different sizes of kitchenware, thickness measures, shapes and portion sizes. Pictures showed different traditionally consumed species with names available in English and the appropriate Inuit language to improve accuracy. Food use information was entered using Epi Info (CDC) and data were double verified (2007) or double entered (2008). Nutrient composition of foods was determined using the Canadian Nutrient File (33). An additional in-house food file was used for foods not on the Canadian Nutrient File; nutrients for these foods were obtained using food labels, recipes and other resources found on the Internet (nutrient values from the US were checked for possible fortification differences with Canadian products).

Using the food use data derived from the Inuit Health Survey, combined with nutritional information of key country foods, we estimated the potential impacts on nutrient intake under the hypothetical scenario that the consumption of primary food species decreased by 50%. We analyzed regional variation of country foods reported to be less abundant over the past 12 months, examined the impact of nutrient intake on Inuit diet with a focus on micronutrients when less of these foods were available, and proposed alternate country food options as potential replacements. Potential alternate country food species based on the feasibility of the substitution in terms of comparable nutrient composition were explored. The proposed substitutions are theoretical choices, derived from the presence of the species in the regional diet at the time of the survey.

## Results

A total of 2,595 adults 18 years and older participated in the Inuit Health Survey encompassing ISR, the 3 regions of Nunavut (Baffin, Kivalliq, Kitikmeot) and the region of Nunatsiavut.

### Country foods less abundant over the past 12 months in all regions

**Fig. 2:**
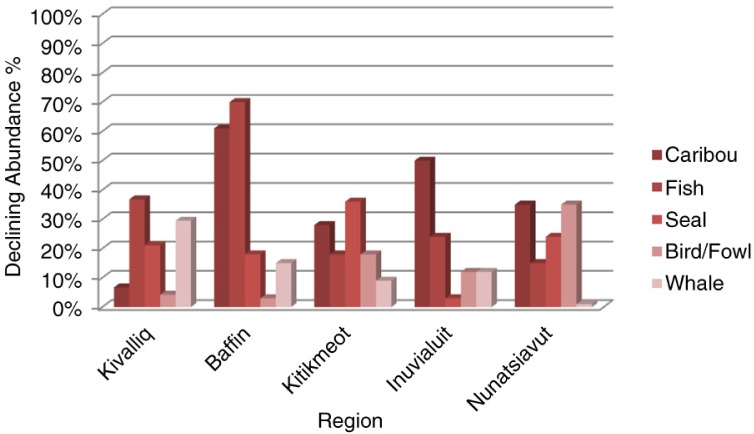
Country foods reported as being less abundant over the past 12 months, per region.

The results show considerable regional variation among country foods found to be less abundant over the past 12 months (Fig. 2). In summary, caribou was the most common response for ISR, Nunatsiavut and the Baffin region of Nunavut. In the Kivalliq and Kitikmeot regions of Nunavut, fish and seal were the most common responses, respectively.

In the Kivalliq region of Nunavut, 37% of the participants stated that fish has become less abundant in the previous year closely followed by whale. Participants also reported seal as being less plentiful. Sixty-one percent of Baffin respondents in Nunavut reported caribou to be less abundant in the previous 12 months, and approximately 18 and 15% of participants stated that seal and whale, respectively, were less plentiful over the previous year. In the Kitikmeot region of Nunavut, seal was identified as the top country food to be less plentiful. Approximately 28% of the survey respondents stated that caribou had become more difficult to find and 18% reported fish as having become scarce. In the ISR, caribou was identified by half of the survey respondents as being less abundant over the past 12 months. In addition, 23% reported fish and whale as having become more difficult to obtain. In Nunatsiavut, 35% of the respondents found that caribou and birds were less abundant in the previous year.

### Nutrient intakes from country foods in all regions

**Fig. 3:**
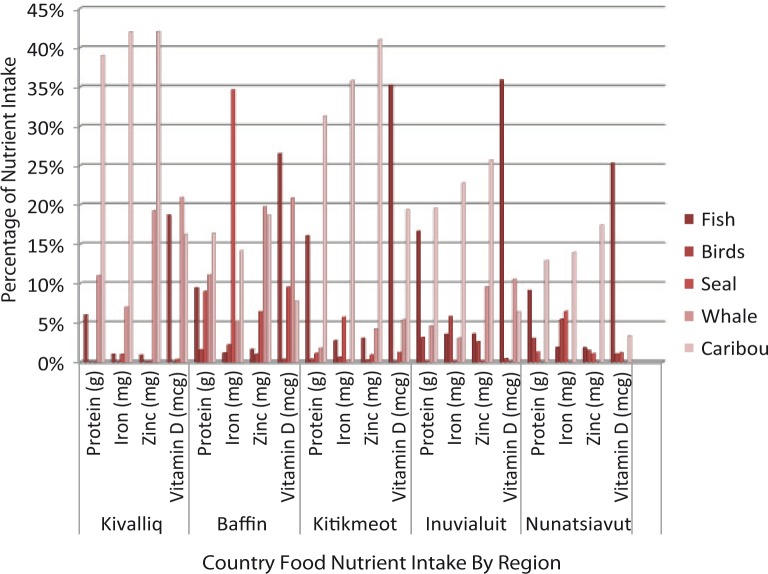
Contribution of nutrient intake to the overall diet from country foods, by region.

Consumption of country food contributed significant nutrition requirement in all studied regions (Fig. 3). In the Kivalliq region of Nunavut, fish contributed less than 2% of all calories but provided almost 19% of vitamin D. Whale supplied high levels of zinc and vitamin D to the diet at 19 and 21%, respectively, and contributed almost 6% to the overall caloric intake. In the Baffin region of Nunavut, caribou provided 5% to the overall caloric intake of the population, and contributed 14 and 19% of iron and zinc, respectively. Seal contributed only 3% to the overall caloric intake, and 9% to protein but it provided a substantial 35% of iron. Whale contributed approximately 20% of both zinc and vitamin D to the overall diet.

In the Kitikmeot region of Nunavut, seal provided almost 6% of iron intake despite limited contribution to the overall caloric intake. Caribou contributed approximately 10% to caloric and fat intake, 19% of daily requirements of vitamin D, and over 30% of protein, iron and zinc. In the ISR, caribou provided approximately 20% of protein intake and over 22% of iron and zinc. Fish contributed approximately 6% to the total caloric intake, about 17% of protein intake and 36% of vitamin D intake. Whale, predominately beluga, contributed only 3% to the total caloric intake but contributed 10% of vitamin D. In Nunatsiavut, caribou contributed approximately 12% of protein, iron and zinc to the total regional diet. Bird intake, which included partridge, duck and goose, made a small contribution to the overall caloric intake as a result of low availability of these species in the region, with only 3% contribution to protein, and approximately 6% contribution to iron intake.

### Nutrient replacement of key country food species per region

Based on the reported observation in declined harvest of food species in each region, we estimated the impacts of a 50% reduction in harvest of 2 species in each region and explored whether it was possible to substitute the food (gram-for-gram) with an alternative country food in the region. The results are presented in Table I.

**Table I:** Potential decrease in nutrient uptake in each region under the hypothetical scenario that the top 2 food species decrease by 50%

	50% decrease in country food intake – Possible nutrient replacements								
	
	Person/day	Food wgt (G)	Calories (K)	Protein (G)	Fat (G)	Carb (G)	Iron (MG)	Zinc (MG)	Vit D (MCG)
Kivalliq									
If **fish** ↓ 50%	**64**	**13,000**	**18,015**	**2,896**	**650**	**0**	**75**	**73**	**447**
Replaced by Duck		13,000	21,590	3,900	507	0	1,371	383	26
If **whale** ↓ 50%	**43**	**12,451**	**27,992**	**2,874**	**1,638**	**0**	**290**	**786**	**250**
Replaced by Goose		12,451	34,666	3,498	2,158	0	925	428	20
Baffin									
If **caribou** ↓ 50%	**102**	**21,909**	**40,731**	**6,357**	**1,495**	**4**	**1,217**	**1,107**	**165**
Replaced by Goose		21,909	60,997	6,156	3,797	0	1,627	753	36
If **seal** ↓ 50%	**61**	**12,731**	**24,497**	**3,490**	**1,033**	**1**	**2,971**	**381**	**203**
Replaced by Ptarmigan		12,731	18,948	3,532	454	0	1,069	189	NV
Kitikmeot									
If **seal** ↓ 50%	**3**	**775**	**1,353**	**249**	**33**	**0**	**208**	**27**	**13**
Replaced by Goose		775	2,157	217	134	0	57	27	1
If **caribou** ↓ 50%	**82**	**22,078**	**44,437**	**6,956**	**1,637**	**3**	**1,304**	**1,195**	**208**
Replaced by muskox		22,078	23,202	4,194	441	NV	993	529	NV
Inuvialuit									
If **caribou** ↓ 50%	**39**	**9,703**	**18,186**	**3,153**	**531**	**0**	**572**	**543**	**60**
Replaced by Moose		9,703	13,535	2,585	279	0	378	562	NV
If **fish** ↓ 50%	**38**	**9,329**	**17,921**	**2,681**	**710**	**19**	**88**	**75**	**337**
Replaced by goose		9,329	25,973	2,621	1,617	0	693	320	15
Nunatsiavut									
If **caribou** ↓ 50%	**23**	**4,680**	**8,341**	**1,585**	**178**	**0**	**284**	**272**	**24**
Replaced by Rabbit		4,680	5,444	1,199	78	0	243	108	2
If **birds** ↓ 50%	**16**	**1,241**	**2,042**	**371**	**49**	**0**	**110**	**23**	**7**
Replaced by Trout (gram-for-gram)		1,241	1,529	252	47	0	6	6	55

Potential alternative food species with comparable nutrient composition are listed as possible substitutes in each region.

A total of 128 participants in the Kivalliq region of Nunavut consumed 26 Kg of fish per year. Therefore, a 50% reduction resulted in 64 persons consuming half of the fish at 13,000 g (Table I). In the Kivalliq region fish provided a significant amount of vitamin D to Inuit diet yet it has been identified as one of the species to be less available in the past 12 months. If duck were used to replace 50% of fish intake, gram-for-gram, far more iron (20X) and zinc (5X) would be consumed however vitamin D intake would be decreased by 94%. Similarly, if whale consumption were to decrease by 50%, it would represent an average loss of 18 mg of zinc per person per day, and approximately 6 µg of the daily requirements of vitamin D. In the event that 50% of current whale consumption was to be replaced with goose, more iron (3X) would be consumed; however, goose meat would supply only half the amount of zinc, and vitamin D intake would decrease by 92%.

Sixty-one percent of Baffin respondents stated that caribou was less abundant in the previous 12 months. While caribou contributed only 5% of caloric intake in the population, it provided 14% of iron and 19% of zinc. Potential substitutes for caribou could be goose, duck, ptarmigan and rabbit. For example, if goose was replaced gram-for-gram for half of the food weight contributed by caribou, fat contribution would more than double and zinc and vitamin D levels would be substantially reduced compared to that provided by caribou. Seal in the region contributed only 3% to the caloric intake and a nominal 9% to protein intake, but supplied a substantial 35% of iron intake. Gram-for-gram replacement of seal with ptarmigan meat would provide less than half the amount of iron and zinc compared to that provided through seal consumption. Vitamin D values were not available for ptarmigan.

Seal, the most commonly reported food species to be less available in the previous year, was little consumed in the Kitikmeot region, contributing less than 1% of total caloric intake, however seal can potentially provide 6% of total iron intake. Gram-for-gram replacement of seal meat with goose meat (with and without skin) would replace only 28% of the iron lost if seals further decreased in availability. Caribou contributed approximately 10% of caloric and fat intakes, 19% of the daily requirement of vitamin D and over 30% of protein, iron and zinc. A 50% decrease in caribou availability would have a substantial impact on the regional diet if not replaced by equally nutrient-dense food sources. Meat from other land mammals in the region, such as muskox, rabbit and ptarmigan, as well as goose and duck could be promoted as alternate species. For example, if caribou was to be replaced by muskox it would contribute approximately 50% of the calories, 76% of iron and 44% of zinc. Vitamin D values were not available for muskox.

Inuvialuit respondents reported caribou and fish to be less available over the past year. For caribou that was consumed it contributed approximately 20% of protein intake and over 22% of iron and zinc intakes. If caribou availability was to decrease by 50%, alternate protein, iron and zinc food sources would have to be consumed to maintain current levels. Land mammals such as muskox and rabbit provide gram-for-gram nearly equal amounts of iron; however, all land mammals mentioned to date with the exception of moose provide less zinc and substantially lower levels of vitamin D (where values were available).

The nutritional importance of fish to the residents of Inuvialuit, in terms of vitamin D intake, was essential. While contributing about 6% to daily caloric intake and 17% of protein intake, fish contributed 36% of vitamin D. While land species may provide a sufficient replacement in terms of iron and zinc contributions, as stated earlier, these foods do not provide adequate amounts of vitamin D.

In Nunatsiavut, 35% of Nunatsiavut respondents reported that caribou was less abundant over the past year but provided over 12% of protein, iron and zinc intake to the overall regional diet. If there was a further decrease in caribou availability, potential regional substitutions could include partridge, duck, goose and rabbit. However, the nutrient contribution of rabbit compared to what would be lost if caribou availability were to decrease by 50%, on a gram-for-gram basis, rabbit provided fewer macronutrients as well as less iron, zinc and vitamin D, making caribou meat far more superior. Approximately 35% of survey respondents stated that birds were also less abundant in the previous year. Bird intake, which included partridge, duck and goose, made a small contribution to the caloric intake, only 3% of protein and approximately 6% of iron intake. Replacement of birds with trout would provide less iron and zinc, but provide more vitamin D on a gram-per-gram basis.

## Discussion

There is no longer any debate about whether or not climate change is having an impact on the Canadian Arctic but rather the extent each region is being affected by it. Results from the comprehensive Inuit Health Survey along with other studies conducted throughout the Arctic indicated that each region is experiencing varying levels of climate-related impacts and opportunities, and a number of communities are already employing various adaptations to deal with those changes (5,22,29–31). Some of those adaptations include the development of youth–elder mentor programs to transmit traditional knowledge, changing the mode and timing of hunts, increase the use of community freezers to store and promote accessibility to country foods, formation of inter-community sharing networks of country foods, and substitution of a declining species by new or alternate species (22,26,30,31).

Similar to other studies (22,30,31), species normally harvested such as caribou, fish, seal, whale and native bird species had been reported as being less abundant to varying degree in each region over the previous 12 months. Hypothetical substitutions of these species with potential alternate species showed that in some instances the nutrient content was similar to that of the declining species, and in other instances the nutrient content was far less superior. For example, marine mammals and fish are the principal sources of vitamin D in the Inuit diet so turning to substitution species such as goose, duck or seal do not compare in terms of vitamin D content. If both species are reported to be less abundant, as was the case in the Kivalliq region in Nunavut, this can have a significant negative impact on the nutritional quality of Inuit diet, and contribute to a broad range of health outcomes (34). Inuit have limited exposure to sunlight throughout the year, and a high prevalence of lactose intolerance has been reported among this population (35). Ensuring access for Inuit to vitamin D–rich country foods or developing feasible nutrition supplement programs would therefore be highly advantageous. In comparison, a land species such as caribou which has been reported to be less abundant over the previous year in all regions except Kivalliq, comparable substitutions have been found in species such as muskox, moose and goose in terms of iron and protein contents. In either case, looking to species substitution as a viable option to ensuring the integrity of Inuit diet is an important type of adaptation to a species decline, and one that has been used in the past. Alternatively, if newer species are not harvested, more expensive and less nutritionally dense store-bought foods may be sought. Recent results from the Inuit Health Survey reported a significant decrease in energy contribution from country food, and a significant increase in market food consumption over time (36). Foods such as sugar-sweetened beverages, chips and pasta all increased in the percentage of total energy consumed among Inuit (36). The fact that adults living in food insecure households had lower frequency of country food intake and obtained more energy from high-sugar foods (17), promoting hunting and harvesting activities along with the consumption of new or alternate species, would be highly beneficial.

The general effects of climate change such as unpredictable weather, a change in large wildlife species distribution and health, and a thinner unstable ice warrant regional approaches to adaptation as they are regional in nature. However, it is important to recognize that many other challenges such as food preferences, local vegetation, appearance or disappearance of smaller migratory wildlife species and the level of consumption of country food are community-specific and it is the level at which adaptation will ultimately take place (31). For example, if one community is more open to hunting and eating new species then that community has an adaptive capacity compared to a community who is less open to it. Interestingly, a study in Alaska used a predictive framework to assess whether 19 rural Alaska Native communities used a switch prey coping strategy in the face of fluctuations in the availability of traditional foods (37). They found that between 1993 and 2004 these communities rarely substituted one resource for another. They pointed out that this may be due to possible complicating factors such as having greater access to market foods and increased formal employment, as well as highlight the need for improved data collection on harvests, hunter effort and socioeconomic characteristics to inform comprehensive models. Another study collected social network, harvest and economic data in 2 Alaska Native Inupiat communities, compared patterns to existing data across 3 decades and found that those highly engaged in market activities were also disproportionately involved in subsistence activities, sharing and cooperation (38). Their study showed that although the communities were dependent on mixed economies (traditional and market), they were not inevitably headed towards full market dependence, and continued to maintain their strong cultural identities in spite of the many challenges Alaska Native communities faced over the past 30 years.

At the regional level, adaptations such as increasing public education around species substitution in terms of building skills and awareness, promoting regional sharing networks and maintaining an up-to-date nutritional monitoring data are just some of the potential solutions to addressing the nutritional burden of Inuit diet: a burden that includes a nutritional transition towards more market foods which are often unhealthy, and the high prevalence of food insecurity experienced by Inuit in all regions (16).

Potentially developing a co-management agency involving all Inuit regions may also be useful. Collaboration with and among communities, researchers and decision makers becomes essential when dealing with a large scale problem like climate change. A co-management agency on food security as it pertains to climate change would enable organizations such as the Hunters and Trappers Organizations and other community groups to provide local insight and Indigenous knowledge to regional/territorial agencies (39). An agency that incorporates these important factors would help bridge the gap at multiple levels from community members to research to territorial and federal decision makers. Bringing together information from all levels, community, science and policy, will potentially increase Arctic food security as climate change continues to exert its effects in the coming decades. Loring and Gerlach (40) recently brought up an interesting point in their meta-analysis of literature review of food security in the North American North: there are many reasons why people are food insecure and many of these challenges have been brought up in this paper; however, they identified governance and policy challenges as the primary drivers of food insecurity. Their recommendations for future research included an improved focus on participatory research and food security interventions that acknowledge and focus on supporting the rights of local peoples to pursue food security on their own terms.

## Conclusion

For Inuit, access to country food means having sufficient, nutritionally adequate and socially acceptable food that is at the core of preserving the integrity of Inuit culture and Inuit health (6). Affordable perishable market foods will certainly play a role in ensuring future food security, but it will not replace the superior nutrients that country foods provide in addition to having an impact on physical health, psychological and social functioning, and on the continuity of Inuit culture (6). Given the superior quality of country foods compared to most available market foods and their association with higher food security, promoting traditional activities and increasing access to country foods may potentially ensure nutritional integrity of Inuit diet, and in turn the health of Nunavummiut.
